# An Elderly Patient With Lumbar Spondylodiscitis and Psoas Abscess: Diagnostic Considerations in the Context of Aerococcus urinae

**DOI:** 10.7759/cureus.84752

**Published:** 2025-05-24

**Authors:** Julian Müller-Kühnle, Moritz Schanz, Severin Schricker, Jörg Latus, Leonie Kraft

**Affiliations:** 1 Department of General Internal Medicine and Nephrology, Robert Bosch Krankenhaus, Stuttgart, DEU

**Keywords:** aerococcus urinae, conservative management, psoas abscess, spondylodiscitis, urinary tract infection

## Abstract

*Aerococcus urinae* is an emerging Gram-positive uropathogen increasingly detected in elderly patients with structural abnormalities of the urinary tract. Although often dismissed as a contaminant, recent evidence highlights its potential to cause invasive infections, including infective endocarditis and, in rare cases, vertebral osteomyelitis or spondylodiscitis. Few such spinal infections have been reported to date.

We describe the case of an 83-year-old man with a history of resected penile carcinoma (pT1a, R0; partial penectomy and bilateral inguinal lymphadenectomy in 2016), and chronic bladder outlet obstruction with associated diverticula, who presented with leukocytosis, elevated inflammatory markers, hypotension, and progressive functional decline.

Initial contrast-enhanced CT imaging revealed lumbar spondylodiscitis at the L2/L3 level, with an associated left-sided psoas abscess. MRI of the lumbar spine subsequently confirmed the diagnosis and showed no evidence of intraspinal extension. While repeated blood cultures remained negative, two sequential urine cultures yielded high-count *A. urinae*; *Enterococcus faecalis* was additionally isolated from the second urine sample. In light of the patient’s advanced age, moderate frailty, and clear clinical improvement under antimicrobial therapy, surgical intervention was not pursued. He was managed without surgical intervention, receiving intravenous piperacillin/tazobactam followed by oral ciprofloxacin, which resulted in clinical stabilization and declining inflammatory markers.

At the first follow-up visit, 10 days after hospital discharge, CRP had decreased substantially (3.9 mg/dL), and the patient’s general condition had improved. At a second follow-up appointment, seven weeks after discharge, CRP had further declined to 1.5 mg/dL, leukocyte count had normalized (6.3 × 10⁹/L), and the patient reported significant pain relief. Antimicrobial therapy was completed as planned, and a further clinical evaluation is scheduled in three weeks at the trauma surgery outpatient clinic.

This case highlights the diagnostic challenges of spinal infections in elderly patients, especially when urine cultures yield multiple potential pathogens. While blood cultures are typically considered more indicative of hematogenous spinal infections, the repeated high-count isolation of *A. urinae* from urine - combined with consistent clinical and radiological findings, and the exclusion of other sources - supports its role as the presumptive pathogen in this case. Increased clinical awareness of *A. urinae* as a potentially invasive organism - even in the absence of bacteremia - is warranted, particularly in severely frail elderly patients for whom invasive diagnostics or surgical interventions may not be feasible.

## Introduction

*Aerococcus urinae* is a Gram-positive, alpha-hemolytic coccus that may appear microscopically similar to *Streptococcus*, *Staphylococcus*, or *Enterococcus* species, often leading to misidentification - particularly in laboratories lacking access to advanced diagnostic tools [1,2]. Long considered a low-virulence commensal or contaminant, *A. urinae* has increasingly been recognized as a clinically relevant pathogen, associated with invasive infections such as bacteremia, infective endocarditis, and, more rarely, musculoskeletal infections including vertebral osteomyelitis and spondylodiscitis [3-10].

The introduction of matrix-assisted laser desorption ionization time-of-flight mass spectrometry (MALDI-TOF MS) has markedly improved the accuracy of species-level identification, contributing to the rising detection of *A. urinae* as a uropathogen - particularly in elderly male patients with structural or functional urinary tract abnormalities [3,9]. Nevertheless, evidence on deep-seated infections remains scarce. Fewer than a dozen cases of *A. urinae*-associated spondylodiscitis have been reported, and optimal diagnostic and therapeutic approaches are yet to be clearly defined [3,5,11,12].

The diagnostic challenge becomes even more pronounced when *A. urinae* is isolated alongside other uropathogens, such as *Enterococcus faecalis*, a well-established cause of urinary and systemic infections.

Here, we report the case of an elderly man with a history of resected penile carcinoma, presenting with lumbar spondylodiscitis and a concomitant left-sided psoas abscess, in whom *A. urinae* was repeatedly isolated in high-count urine cultures (>100,000 CFU/mL). Despite negative blood cultures and the absence of direct microbiological sampling from the spinal lesion - a known limitation - the clinical constellation, including radiological and laboratory findings, and the lack of alternative infectious foci, supported a possible urinary-source spinal infection. Notably, several published cases of *A. urinae*-associated spondylodiscitis have similarly reported negative blood cultures, underscoring the diagnostic relevance of urine isolates in this context. This case highlights the importance of integrating microbiological, imaging, and clinical data in severely frail, elderly patients to support a pragmatic and targeted therapeutic decision-making process.

## Case presentation

An 83-year-old man with a history of penile carcinoma (pT1a, R0; cT3), treated with partial penectomy in March 2016 and bilateral laparoscopic inguinal lymphadenectomy in April 2016, was admitted for evaluation of progressive functional decline, reduced oral intake, and right-sided arm pain following a domestic fall three weeks prior.

There was no history of urethral stricture, but, due to the altered anatomy, bladder catheterization had previously required specialist placement. His past medical history included arterial hypertension and type 2 diabetes mellitus.

Prior to the fall, the patient had been living at home with his wife, independently mobile with a walker. However, due to progressive immobility and increasing care needs, ambulatory nursing support had been initiated one week before admission. A formal care level (Pflegegrad) had already been applied for. At the time of presentation, he was unable to walk unassisted and demonstrated clinical features consistent with moderate frailty (Clinical Frailty Scale: 6), with gradual improvement noted during the hospital stay and after discharge.

According to the patient, he had lost balance while attempting to get into bed and had fallen onto his right side. There were no signs of syncope, loss of consciousness, abnormal movements, or head trauma. He initially remained at home but was referred for inpatient evaluation by his general practitioner, due to increasing right upper arm pain.

On admission, blood pressure measured in a seated position on the right arm was 88/67 mmHg. The patient was afebrile (36.8°C), with a heart rate of 80 bpm, and oxygen saturation of 97% on room air. He appeared clinically frail, though without signs of delirium or cognitive impairment. Nutritional status was preserved. No signs of meningism or tremor were observed. Muscle tone was increased in the thighs and right upper arm.

The cardiopulmonary and abdominal examinations were unremarkable. Bilateral, non-pitting pretibial edema was present, more pronounced on the right side. There were no signs of chronic venous insufficiency or localized inflammation. Neurological evaluation revealed moderate right-sided arm and leg weakness. In the upper limb, no active antigravity movement was possible (Medical Research Council, or MRC grade 2), likely pain-related. The right leg showed holding weakness (MRC grade 3), although examination was limited due to restricted mobility. The left iliopsoas muscle also demonstrated weakness (MRC grade 3), while cranial nerve and sensory functions were preserved.

Musculoskeletal examination revealed localized tenderness over the proximal right humerus (bicipital groove), with restricted active, but preserved passive, range of motion in the right shoulder. A superficial hematoma was present over the mid-upper arm. There were no signs of joint effusion or neurovascular compromise.

Laboratory results showed leukocytosis (29,000/μL), elevated CRP (16.2 mg/dL), and procalcitonin (1.47 ng/mL), consistent with systemic inflammation. Serum creatinine was elevated (1.89 mg/dL; eGFR 32 mL/min/1.73 m²), with mild metabolic acidosis (pH 7.27). Serum albumin was reduced (2.85 g/dL). Urinalysis revealed marked leukocyturia (>100 WBCs/μL), erythrocyturia, and significant bacteriuria.

Initial imaging included a shoulder X-ray (no fracture), cranial and cervical CT (no acute pathology), and abdominal ultrasound (no abnormalities). The bladder was well filled with a regular wall; no free fluid was noted.

**Figure 1:**
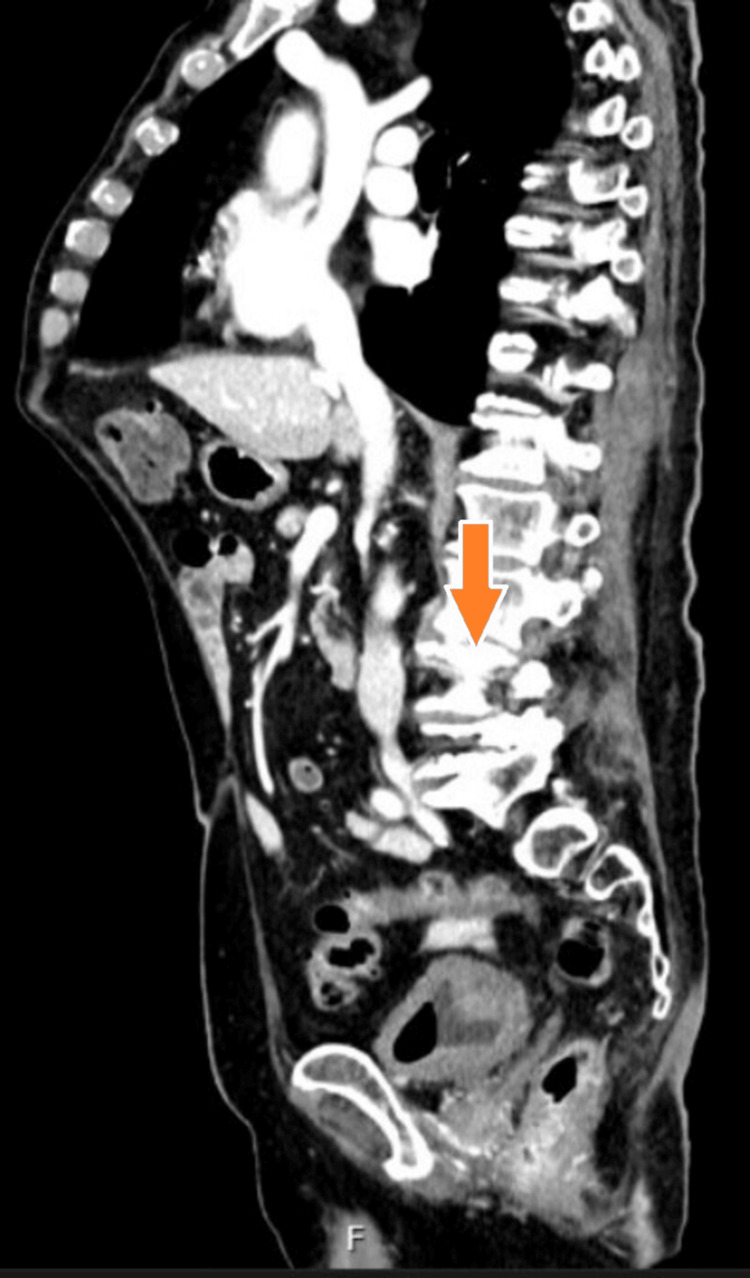
Sagittal CT scan of the lumbar spine demonstrating destructive changes at the L2/L3 intervertebral space, consistent with spondylodiscitis. The orange arrow highlights the area of vertebral involvement.

**Figure 2:**
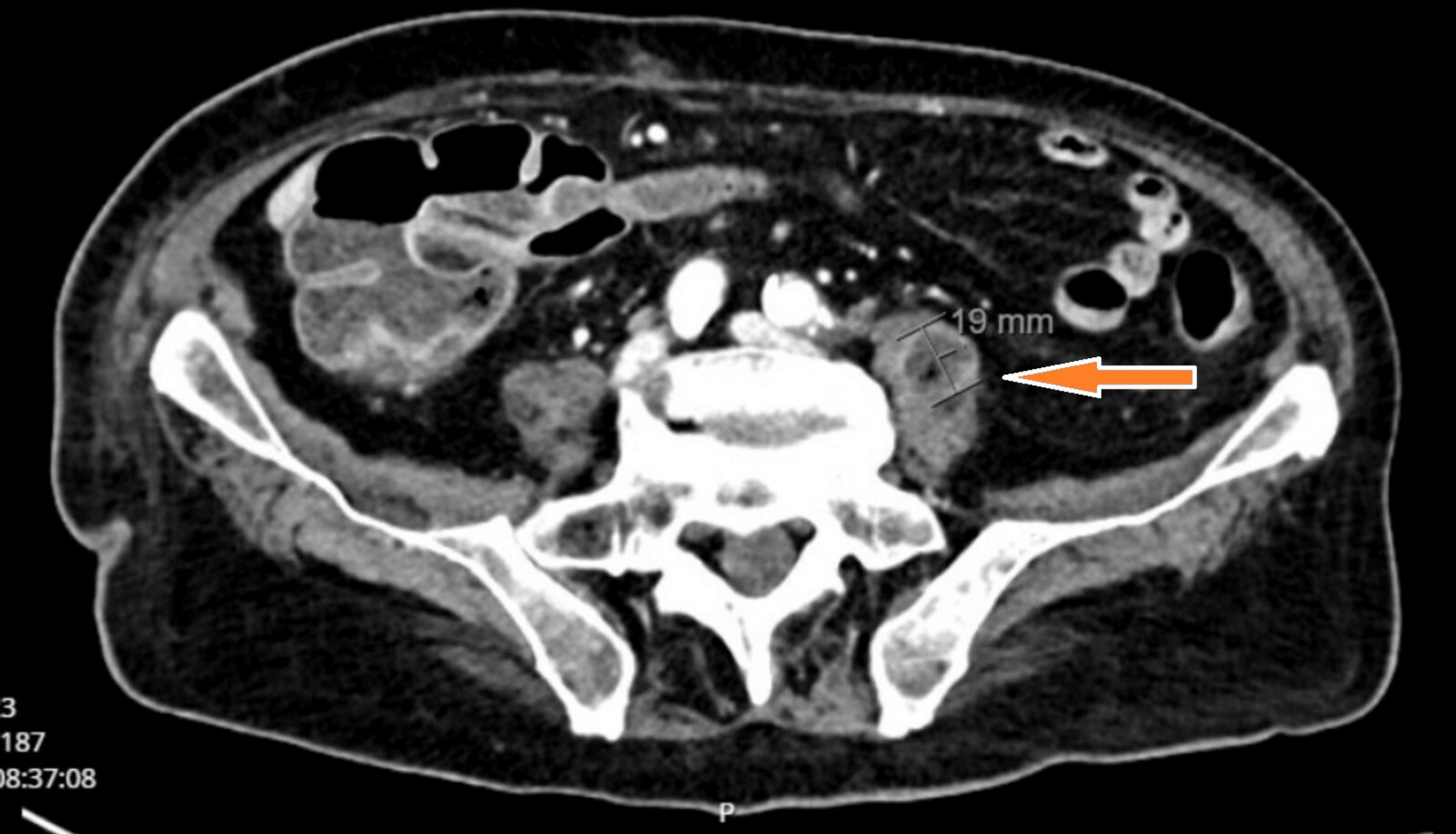
Axial CT scan at the L3/L4 level demonstrating a left-sided psoas abscess adjacent to the affected vertebral bodies. The orange arrow highlights the abscess cavity.

**Figure 3:**
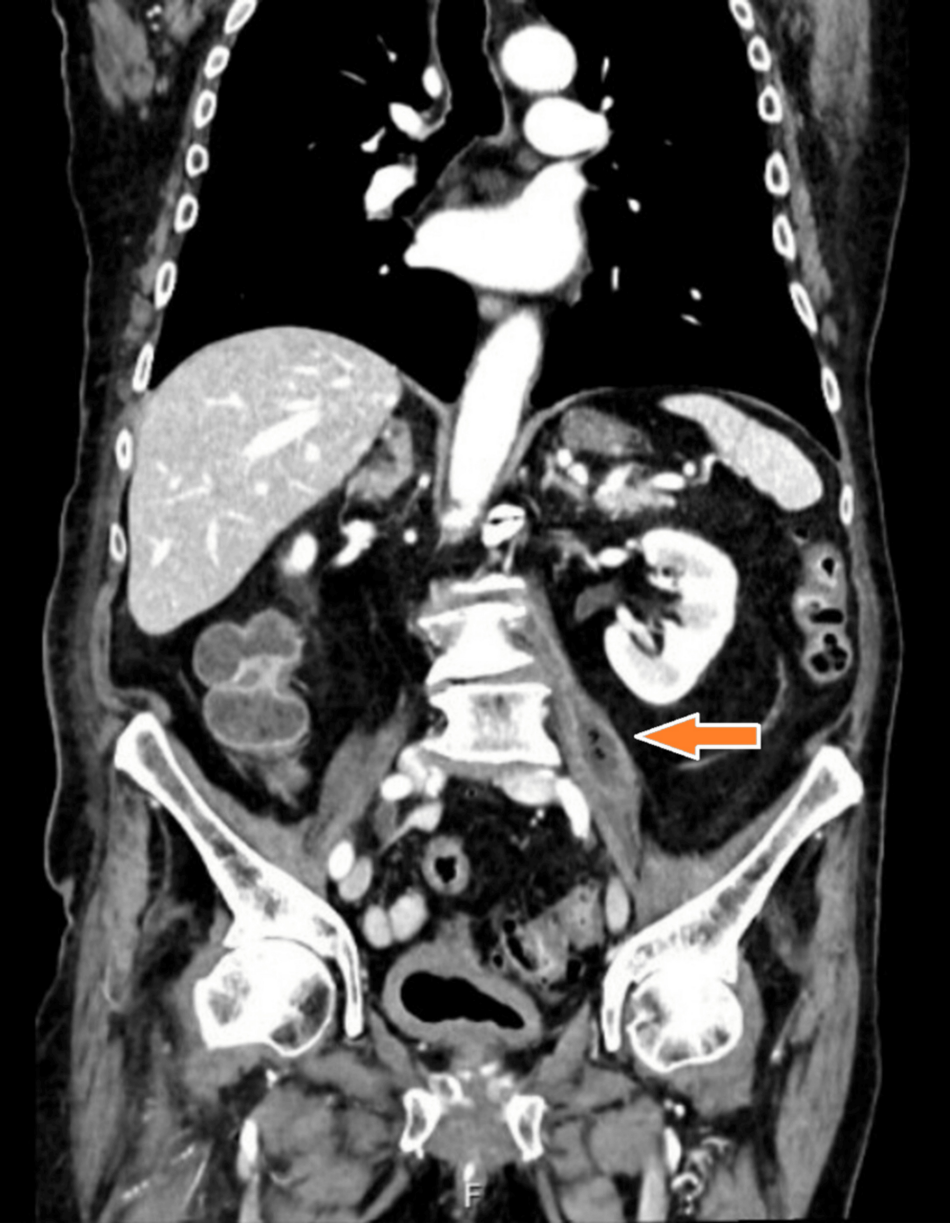
Coronal CT scan of the abdomen and pelvis demonstrating a hypodense, elongated fluid collection within the left psoas major muscle (orange arrow).

Given persistent symptoms and elevated inflammatory markers, contrast-enhanced CT of the abdomen was performed. It revealed a left-sided psoas abscess (8.2 × 1.9 cm), extending toward the L3/L4 intervertebral disc (Figures 1-3).

**Figure 4:**
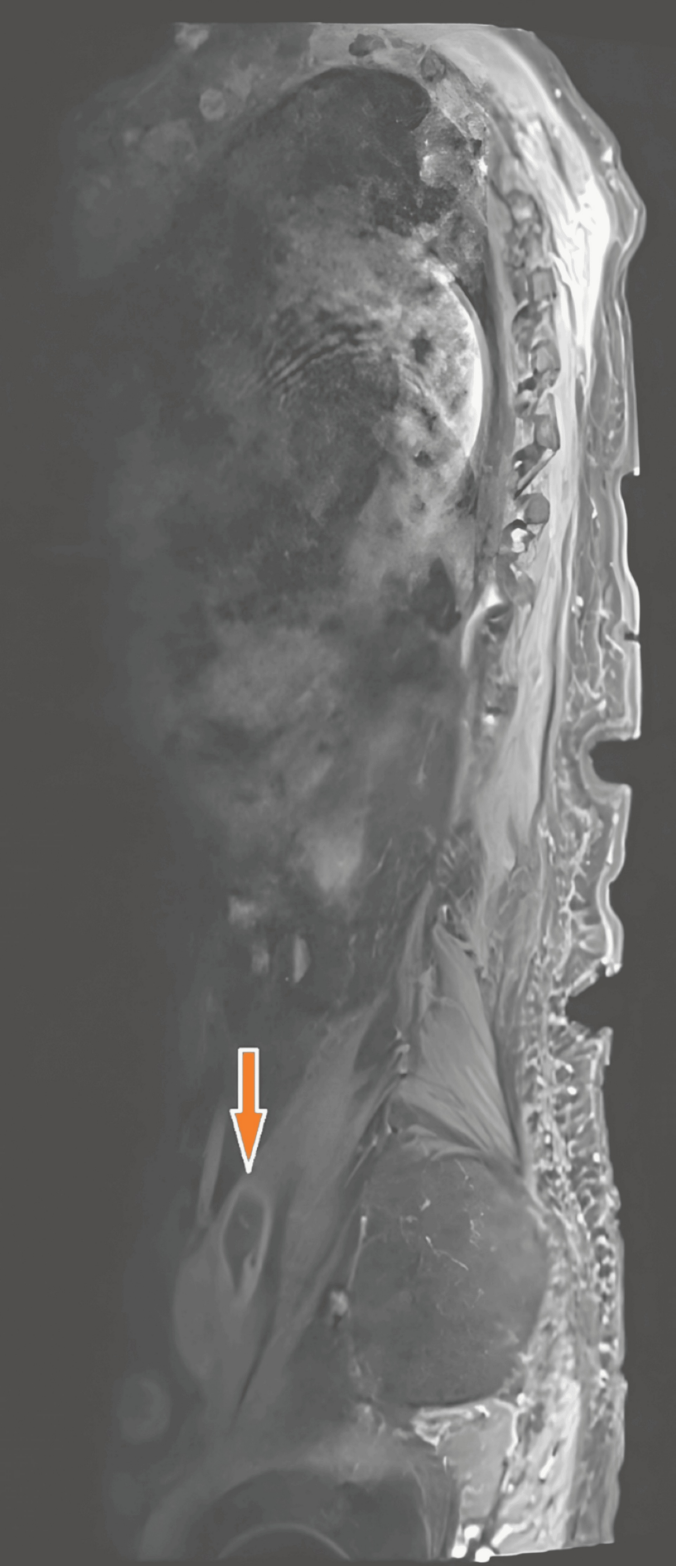
Sagittal MRI of the lumbar spine demonstrating a hyperintense fluid collection along the left psoas muscle, consistent with a psoas abscess. The orange arrow highlights the abscess cavity.

**Figure 5:**
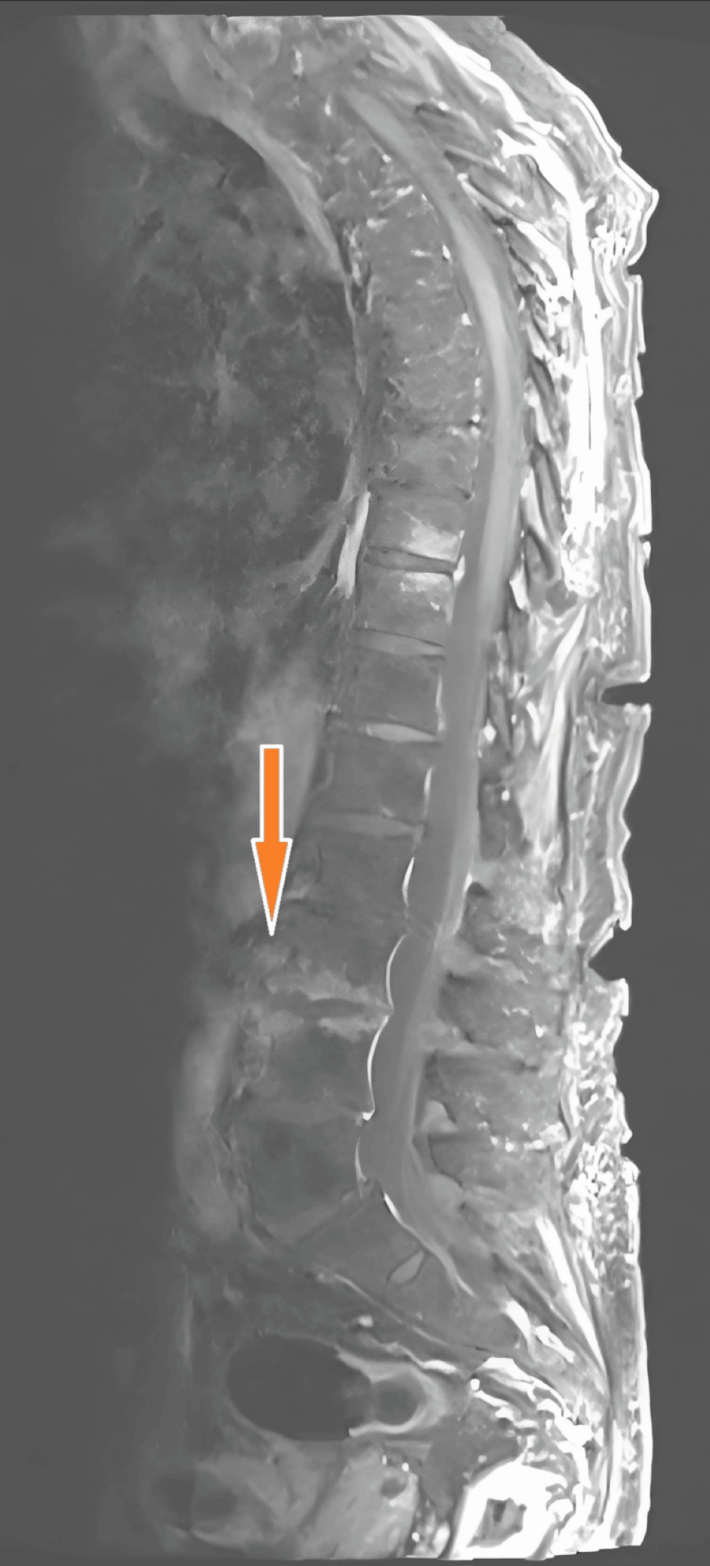
Sagittal MRI of the lumbar spine showing inflammatory changes and destruction at the L2/L3 intervertebral space, consistent with spondylodiscitis. No intraspinal abscess formation was observed. The orange arrow indicates the site of vertebral involvement.

MRI of the lumbar spine (T1-weighted Turbo Spin Echo, or T1-TSE: 3 mm) confirmed florid spondylodiscitis at L2/L3, with adjacent soft tissue involvement, but no intraspinal extension (Figures 4-5).

Microbiological workup revealed two sequential urine cultures: the first showed monomicrobial *A. urinae* at 100,000 CFU/mL. The second urine culture identified both *A. urinae* and *E. faecalis*, each at 100,000 CFU/mL. Two sets of blood cultures (aerobic and anaerobic) were obtained during the first 48 hours of hospitalization and remained negative throughout. The *A. urinae* isolate was susceptible to penicillins, carbapenems, vancomycin, rifampicin, and fluoroquinolones.

Urological evaluation confirmed bladder outlet obstruction due to benign prostatic hyperplasia, with associated bladder diverticula. A transurethral catheter was placed for immediate decompression after ultrasound demonstrated a post-void residual volume exceeding 800 mL, confirming significant urinary retention. Cystoscopy and bladder scan were not performed acutely.

Empirical therapy with ceftriaxone was escalated to intravenous piperacillin/tazobactam following CT confirmation of spondylodiscitis and psoas abscess. After susceptibility testing and clinical stabilization, therapy was de-escalated to oral ciprofloxacin (500 mg BID), with a planned total antibiotic course of six weeks.

Given the patient’s comorbidities, complex anatomical situation, and favorable clinical response to antimicrobial therapy, both surgical intervention and image-guided drainage were deferred. Interventional radiology also declined percutaneous drainage due to the close proximity of vascular and neural structures. The patient was managed non-surgically and transferred to a trauma surgery ward for inpatient rehabilitation.

At the first follow-up visit (10 days after discharge), CRP had decreased to 3.9 mg/dL, and the patient had regained the ability to ambulate with a walker. A second follow-up appointment, seven weeks post-discharge, showed further clinical improvement: a CRP of 1.5 mg/dL, normalized leukocyte count (6.3 × 10⁹/L), and cessation of antimicrobial therapy. A further clinical review was scheduled in the trauma surgery outpatient clinic.

## Discussion

*A. urinae* is an increasingly recognized, yet often underdiagnosed, pathogen, particularly in elderly patients with structural urological abnormalities. The implementation of MALDI-TOF MS has significantly improved species-level identification, contributing to the growing recognition of *A. urinae* in urinary tract infections as well as invasive diseases such as bacteremia, endocarditis, and - more rarely - spondylodiscitis [3,5,7,9].

In the present case, *A. urinae* was repeatedly isolated from urine cultures, including once as a high-count monomicrobial finding. This coincided with the patient’s clinical deterioration and marked inflammatory response. The absence of an alternative infectious focus, together with imaging evidence of florid spondylodiscitis at L2/L3 and an adjacent psoas abscess, supports *A. urinae* as the likely causative pathogen - despite the lack of microbiological sampling from spinal or paraspinal tissue. Risk factors for invasive aerococcal infection, including bladder outlet obstruction, multiple diverticula, and prior urological malignancy, were also present.

Radiologic findings further support a urinary source of infection. An initial contrast-enhanced CT scan revealed a left-sided psoas abscess in anatomical continuity with the L3/L4 intervertebral disc space (Figure 2), destructive changes in the adjacent lumbar vertebrae at the L2/L3 level (Figure 1), and the full extent of the encapsulated fluid collection within the left psoas major muscle (Figure 3). No air-fluid levels were observed. To confirm and further delineate these findings, a single MRI study of the lumbar spine was performed, which verified spondylodiscitis at L2/L3 (Figure 5) and demonstrated adjacent paraspinal soft tissue involvement without intraspinal extension (Figure 4). This sequence of imaging supports a unifying infectious process involving the urinary tract, retroperitoneal tissues, and lumbar spine.

While hematogenous spread remains the most commonly discussed pathomechanism for spinal infections of urogenital origin, this case also raises the possibility of contiguous extension via the retroperitoneum or regional lymphatic drainage. The anatomical proximity of the bladder and urinary tract to the iliopsoas muscle and lumbar spine - together with the absence of bacteremia and the small size of the psoas abscess - suggests a localized, possibly lymphatic, ascending route of infection. Although no urinary tract extravasation or bladder wall defect was identified on imaging, lymphatic or microscopic retroperitoneal spread remains pathophysiologically plausible and may help explain the absence of systemic dissemination.

Differential diagnoses, such as degenerative spine disease, iatrogenic or post-traumatic spondylodiscitis, and unrecognized bacteremia, were considered but deemed unlikely based on the patient’s history, imaging, and lack of spinal instrumentation or trauma.

The identification of *E. faecalis* in a second polymicrobial urine culture introduced diagnostic ambiguity. While *E. faecalis* is a known uropathogen and occasional cause of spondylodiscitis [13,14], its absence from the initial culture and later appearance - after partial clinical improvement - argue against a primary etiologic role. This likely represents transient colonization or secondary co-isolation. Similar diagnostic pitfalls have been reported: Torres-Martos et al. [6] described a case in which initial treatment targeted *E. faecalis*, but *A. urinae* was later confirmed as the causative agent via disc aspiration.

Importantly, the absence of bacteremia does not exclude a deep-seated aerococcal infection. Several published cases of *A. urinae*-associated spondylodiscitis reported negative blood cultures, with diagnosis established only through spinal or bone tissue sampling [3,11,12]. Degroote et al. [12], for instance, described a patient with sterile blood and urine cultures in whom *A. urinae* was identified via intraoperative bone biopsy. These reports - and our findings - highlight the diagnostic relevance of high-count monomicrobial urine cultures when interpreted in conjunction with clinical and radiological findings, especially in frail patients in whom invasive diagnostics are not feasible.

The susceptibility profile of the *A. urinae* isolate in our case supported the selected antimicrobial strategy. The strain was sensitive to penicillins, carbapenems, vancomycin, and fluoroquinolones, allowing structured de-escalation from intravenous beta-lactam therapy to oral ciprofloxacin. This approach aligns with previous reports and was followed by clinical stabilization, decline in inflammatory markers, and functional recovery [3,5,12].

Treatment durations for aerococcal spondylodiscitis in the literature range from 6 to 28 weeks [3,4,6,7]. Most authors recommend prolonged antibiotic therapy with agents demonstrating adequate bone penetration. In our case, the favorable course, lack of neurological deficits, radiological stability, and relevant comorbidities - including frailty and complex urological anatomy - supported a conservative, non-surgical approach. Interventional radiology was consulted, but deferred drainage due to the anatomical location of the abscess and potential procedural risk.

In summary, this case emphasizes the potential role of *A. urinae* in spinal infections, even in the absence of bacteremia. It underscores the importance of integrating microbiological, radiological, and clinical data, particularly when interpreting polymicrobial urine cultures. While longer-term follow-up beyond seven weeks would strengthen the outcome assessment, the documented clinical and biochemical response supports the effectiveness of conservative therapy in this frail patient with a localized infection.

## Conclusions

Spondylodiscitis with a concomitant psoas abscess represents a diagnostic and therapeutic challenge in elderly patients, particularly when invasive tissue sampling is not feasible. This case underscores the importance of evaluating urologic sources of infection - especially in the presence of structural urinary tract abnormalities and polymicrobial urine cultures. Although *A. urinae* remains a rare cause of spinal infection, growing evidence supports its potential pathogenic role in elderly males with urologic comorbidities. Even in the absence of bacteremia or direct tissue confirmation, repeated high-count urine cultures, compatible imaging findings, and systemic inflammation may justify its consideration as the probable causative agent.

Importantly, this case highlights the need to consider hematogenous, lymphatic, and contiguous routes of spread. The anatomical proximity between the urinary tract, psoas muscle, and lumbar spine - along with localized infection and lack of systemic dissemination - supports a plausible per continuitatem mechanism. Ultimately, this case illustrates the value of a multidisciplinary, individualized approach in multimorbid geriatric patients with complex infections. Early recognition of emerging pathogens such as *A. urinae*, combined with targeted antimicrobial therapy and careful clinical monitoring, may enable successful conservative treatment while avoiding unnecessary surgical risks. Interpreting polymicrobial urine cultures within their clinical context remains essential to guide appropriate management.
